# Definition and Characteristics of Behavioral Medicine, and Main Tasks and Goals of the International Society of Behavioral Medicine—an International Delphi Study

**DOI:** 10.1007/s12529-020-09928-y

**Published:** 2020-09-09

**Authors:** Joost Dekker, Marie Amitami, Anne H. Berman, Helen Brown, Bryan Cleal, Maria João Figueiras, Lila J. Finney Rutten, Egil A. Fors, Konstadina Griva, Jing Gu, Chris Keyworth, Maria Kleinstäuber, Claas Lahmann, Joseph T. F. Lau, Bernd Leplow, Li Li, Hanna Malmberg Gavelin, Ricarda Mewes, Phoenix K. H. Mo, Barbara Mullan, Frank J. Penedo, Judith Prins, Teresa Rodríguez Rodríguez, Sharon A. Simpson, Adrienne Stauder, Martti T. Tuomisto, Deborah Jones Weiss, Urs M. Nater

**Affiliations:** 1 Department of Rehabilitation Medicine and Department of Psychiatry, Amsterdam University Medical Centers, PO Box 7057, 1007 MB Amsterdam, the Netherlands; 2 Community-Based Medicine, Kagoshima University Graduate School of Medical and Dental Sciences, Kagoshima, Japan; 3 Center for Psychiatry Research, Department of Clinical Neuroscience, Karolinska Institutet & Stockholm Health Care Services, Stockholm, Stockholm Region Sweden; 4 Department of Psychology, Uppsala University, Uppsala, Sweden; 5 Institute for Physical Activity and Nutrition, Deakin University, Melbourne, Australia; 6 Steno Diabetes Center Copenhagen, Copenhagen, Denmark; 7 Psychology Department, College of Natural and Health Sciences, Zayed University, Abu Dhabi, United Arab Emirates; 8 Department of Health Sciences Research, Mayo Clinic College of Medicine, Mayo Clinic, Rochester, MN USA; 9 General Practice Research Unit, Department of Public Health and Nursing, Faculty of Medicine and Health Science, Norwegian University of Science and Technology, Trondheim, Norway; 10 Lee Kong Chian School of Medicine, Nanyang Technological University, Singapore, Singapore; 11 School of Public Health, Sun Yat-sen University, Guangzhou, China; 12 Manchester Centre for Health Psychology, Division of Psychology & Mental Health, School of Health Sciences, Faculty of Biology, Medicine and Health, The University of Manchester, Manchester, UK; 13 Department of Psychological Medicine, Dunedin School of Medicine, University of Otago, Dunedin, New Zealand; 14 Department of Psychosomatic Medicine and Psychotherapy, Medical Center, Faculty of Medicine, University of Freiburg, Freiburg, Germany; 15 Centre for Health Behaviours Research, Jockey Club School of Public Health and Primary Care, The Chinese University of Hong Kong, New Territory, Hong Kong, China; 16 Institute for Psychology, Martin-Luther-University Halle-Wittenberg, Halle, Germany; 17 Beijing Friendship Hospital, Capital Medical University, Beijing, China; 18 Academic Unit for Psychiatry of Old Age, University of Melbourne, Melbourne, Australia; 19 Department of Psychology, Umeå University, Umeå, Sweden; 20 Outpatient Unit for Research, Teaching, and Practice, Faculty of Psychology, University of Vienna, Vienna, Austria; 21 School of Psychology, Faculty of Health Sciences, Curtin University, Perth, Australia; 22 Sylvester Comprehensive Cancer Center, University of Miami, Miami, FL USA; 23 Radboud Institute for Health Sciences, Radboud University Medical Centre, Nijmegen, the Netherlands; 24 Science and Technology Department, University Hospital “Dr. Gustavo Aldereguía Lima”, Cienfuegos, Cuba; 25 Cienfuegos Medical School, Cienfuegos, Cuba; 26 MRC/CSO Social and Public Health Sciences Unit, Institute of Health and Wellbeing, University of Glasgow, Glasgow, UK; 27 Institute of Behavioural Sciences, Semmelweis University Budapest, Budapest, Hungary; 28 Faculty of Social Sciences (Psychology), Tampere University, Tampere, Finland; 29 Department of Psychiatry & Behavioral Sciences, University of Miami Miller School of Medicine, Miami, FL USA; 30 Faculty of Psychology, University of Vienna, Vienna, Austria

**Keywords:** Behavior, Medicine, Definition, Goals

## Abstract

**Background:**

In the past decades, behavioral medicine has attained global recognition. Due to its global reach, a critical need has emerged to consider whether the original definition of behavioral medicine is still valid, comprehensive, and inclusive, and to reconsider the main tasks and goals of the International Society of Behavioral Medicine (ISBM), as the umbrella organization in the field. The purpose of the present study was to (i) update the definition and scope of behavioral medicine and its defining characteristics; and (ii) develop a proposal on ISBM’s main tasks and goals.

**Method:**

Our study used the Delphi method. A core group prepared a discussion paper. An international Delphi panel rated questions and provided comments. The panel intended to reach an a priori defined level of consensus (i.e., 70%).

**Results:**

The international panel reached consensus on an updated definition and scope of behavioral medicine as a field of research and practice that builds on collaboration among multiple disciplines. These disciplines are concerned with development and application of behavioral and biomedical evidence across the disease continuum in clinical and public health domains. Consensus was reached on a proposal for ISBM’s main tasks and goals focused on supporting communication and collaboration across disciplines and participating organizations; stimulating research, education, and practice; and supporting individuals and organizations in the field.

**Conclusion:**

The consensus on definition and scope of behavioral medicine and ISBM’s tasks and goals provides a foundational step toward achieving these goals.

**Electronic supplementary material:**

The online version of this article (10.1007/s12529-020-09928-y) contains supplementary material, which is available to authorized users.

## Introduction

The field of behavioral medicine was originally defined at the Yale Conference on behavioral medicine, now more than 40 years ago [1]. Behavioral medicine was defined as “the interdisciplinary field concerned with the development and integration of behavioral and biomedical science knowledge and techniques relevant to health and illness and the application of this knowledge and these techniques to prevention, diagnosis, treatment and rehabilitation” [1]. The definition was developed by a group of outstanding scholars from North America, aiming to increase communications of theory, research, and applications in the field, as well as to facilitate access to research funding [2]. During those 40 years, the field has significantly evolved and expanded, witnessing major scientific discoveries and developments, e.g., the role of depression in cardiovascular disease [3], and the increasing emphasis on behavior change in the prevention of disease [4]. In addition to basic science and clinical practice, the scope of behavioral medicine has since broadened to include public health [5]. Behavioral medicine has attained global recognition. Due to its global reach, the field of behavioral medicine currently faces the challenges and opportunities represented by the many international differences in perspectives on health and disease, implementation in a diversity of health care systems, and engagement across varied scientific traditions [6].

The International Society of Behavioral Medicine (ISBM), a federation of 26 national societies with a shared focus on behavioral science and behavioral medicine, promotes behavioral science, research, education, and practice in a global context, embracing the complexity and diversity of its member organizations. Given the expanded scope and reach of behavioral medicine, a critical need arose to consider whether the original definition of behavioral medicine is still valid, comprehensive, and inclusive. Issues to consider included the meaning of the term “behavior,” the need to refer to “health” in addition to “medicine,” the implications of the increased emphasis on public health, the question whether behavioral medicine is a field or a discipline, the implications of the growing emphasis on the development and evaluation of implementation strategies, and other issues [6, 7] (see also Supplementary file 1).

Development of a preliminary proposal for an updated definition and scope of behavioral medicine [6] led to considerable discussion and debate [8–12], underscoring the need to reconsider the original definition. The need for further input from ISBM members was emphasized [7]. A slightly revised proposal [7] was presented and discussed at the International Congress of Behavioral Medicine 2018, in the context of discussions on the main tasks and goals, and future organization of ISBM. The ISBM Board decided to install the ISBM Taskforce “Definition, Goals and Organization” charged with the task to develop a proposal on ISBM’s main tasks and goals, and to take the updated definition and scope of behavioral medicine and its defining characteristics as a starting point. With the intent to understand and integrate current cross-cultural and cross-disciplinary perspectives on behavioral medicine in a global context, the purpose of the present study was to (i) update the definition and scope of behavioral medicine and its defining characteristics; and (ii) develop a proposal on ISBM’s main tasks and goals.

## Method

### Design

Our study used the Delphi method. The design was derived from a recent review of methods used in Delphi studies [13]. A core group prepared a discussion paper, identified items for discussion, and developed a questionnaire. A Delphi panel rated the questions and provided comments. Subsequently, the core group developed a proposal on how to proceed in the next round of discussion. The panel intended to reach the a priori defined level of consensus (i.e., 70%) in two to three rounds of discussion.

### Panel

The Delphi panel consisted of the core group and representatives of various organizations. Members of the *core group* (AHB, MK, BL, JL, BM, UMN, FP, AS, DJW, and the non-voting moderator/chair JD) were selected by the ISBM Executive Committee. All ISBM member societies, ISBM’s early career network (INSPIRE), and several emerging collaborative groups in the field of behavioral medicine were invited to nominate a *representative* to the panel. The following organizations nominated a representative: American Psychosomatic Society, Australasian Society for Behavioral Health and Medicine, Behavioral Medicine Section of the Finnish Association of Social Medicine, Chinese Society of Behavioural Medicine, Danish Society of Psychosocial Medicine, Division of Behavioural Medicine of the Cuban Society of Health Psychology, German College of Psychosomatic Medicine, German Society of Behavioral Medicine and Behavior Modification, Hong Kong Society of Behavioral Health, Hungarian Society of Behavioral Sciences and Medicine, Japanese Society of Behavioral Medicine, Netherlands Behavioral Medicine Federation, Norwegian Society of Behavioral Medicine, Portuguese Society of Health and Behavior, Society of Behavioural Health Singapore, Society of Behavioral Medicine (USA), Swedish Behavioral Medicine Society, UK Society of Behavioral Medicine, INSPIRE, and emerging collaborative groups on behavioral medicine in China and Thailand (and another representative of an emerging collaborative group, who did not respond in any of the Delphi rounds).

### Discussion Paper

The core group prepared a discussion paper, drawing on previous publications [1, 5–12], previous discussions at the ISBM Governing Council meeting and a session held at the International Congress of Behavioral Medicine (Santiago, November 2018) [14]. The discussion paper is in Supplementary file 1.

### Items for Discussion, Questionnaire, and Procedure

In round 1, based on the discussion paper, the core group defined 9 items for discussion, resulting in 10 questions (a question on each item, and an open question) (see Supplementary file 2). In round 2, the core group defined 3 items for discussion (see Supplementary file 2). In rounds 3 and 4, the core group defined 1 item for discussion (see Supplementary file 2). The Delphi panel (consisting of the core group and the representatives) rated the questions on a 5-point Likert scale (strongly agree, agree, neutral, disagree, strongly disagree). The need for strong input from the panel was emphasized throughout the entire Delphi study. In round 1, the panel members were invited to bring up additional items for discussion. In all rounds, they were encouraged to make proposals on the definition and scope of behavioral medicine, the defining characteristics, and main tasks and goals, and to provide comments on each item/question.

### Questionnaire Rounds, Consensus, and Feedback

The study was designed to have two to three rounds of discussion, depending on how soon consensus would be reached. The a priori consensus level was set at 70% of respondents’ voting in the strongly agree/agree (or disagree/strongly disagree) categories. The moderator summarized the responses in each round and the core group then made proposals on items for further discussion. After each round, a summary of the responses was distributed among panel members. The members explicitly indicated whether they agreed on the summary of the responses: the a priori consensus level of 70% was also used to determine whether the panel agreed with the summary. All study communication was via email.

### Reporting

After the Delphi study was completed and the first draft of the manuscript was available, members of the panel who actually participated in the Delphi study were invited to become a co-author on the present paper. The authors also reported to the ISBM Board and Governing Council.

## Results

### Main Results

The final texts on the definition and scope of behavioral medicine, its defining characteristics, and the proposal regarding ISBM’s main tasks and goals can be found in Boxes 1, 2, and 3, respectively. These results were obtained in four rounds of discussion among the members of the panel, as detailed below.

Box 1 The definition and scope of behavioral medicine

**Table:** 

Behavioral medicine is a field of research and practice that builds on collaboration among multiple disciplines. These disciplines are concerned with the development and integration of behavioral and biomedical knowledge relevant to health and disease. Behavioral knowledge refers to psychosocial, societal, economic, cultural, existential, and environmental processes of health- and disease-related behavior, and biomedical knowledge refers to physiological, pathological, and medical processes. This knowledge is applied to prevention, health promotion, diagnosis, treatment, rehabilitation, and care. The scope of behavioral medicine is broadly inclusive of behavioral and biomedical science as well as clinical and public health practice.	

#### Delphi Panel and Response Rate

The Delphi panel consisted of 29 people: 6 members of the core group only, 22 representing an organization (including 3 who were also core group members), and 1 non-voting moderator. The overall response rate in round 1 was 82% (core group: 78%; representatives: 86%), in round 2: 93% (core group 100%; representatives: 86%), in round 3: 86% (core group: 78%; representatives: 95%), and in round 4: 93% (core group: 100%; representatives: 91%).

#### Round 1

Consensus was reached for 8 out of 9 items in round 1, with the percentage of agreement ranging from 83 to 96%. The one exception concerned the item on the need for a name change: the agreement on that item was 48%. The ratings are listed in Supplementary file 3, Table S1. The degree of consensus and panel member’s comments are summarized below for each item, followed by the core group’s considerations after round 1.

##### Item 1. Definition and Scope of Behavioral Medicine

The panel reached consensus on the proposed definition and scope of behavioral medicine (agreement: 87%). Respondents mentioned that the explanation of “behavioral knowledge” needs to be integrated into the definition, as it refers to a broader understanding of “behavioral” than is usually encountered. In the listing of processes, use of the term “psychosocial processes” was suggested, along with the inclusion of economic and political processes and environmental influences, thereby emphasizing the idea of multiple determinants of health. Respondents questioned whether “techniques” should be mentioned, as the term “application of knowledge” is sufficient to convey the practical application of behavioral medicine. The term “methodologies” was suggested as an alternative to “techniques.” It was also mentioned that the uniqueness of behavioral medicine needs to be emphasized: the aim is not solely to synthesize different perspectives, but to transcend traditional boundaries and thereby create new knowledge. The current definition was felt to be too long and difficult to remember, and several minor edits were proposed. Finally, single suggestions included not removing “etiology” from areas where behavioral medicine can be applied, emphasizing the user perspective, referring to psychophysiology, referring to health services, and use of the term “inter-professional.”

##### Item 2. Behavioral Medicine Is a Field and Not a Discipline

The panel reached consensus regarding characterizing behavioral medicine as a field rather than as a discipline (agreement 96%). The panel relied on Sommer’s characterization of the difference between a field and discipline [15]: “*Discipline.* (…) Members of a discipline have been trained in departments associated with that discipline and study many different phenomena using a shared epistemology.” (p. 2) “*Field of study*. (…) People in a field of study have been trained in various disciplines and professions but all focus on a common problem area.” (p. 2) (see Supplementary file 1). Respondents mentioned that behavioral medicine is frequently thought of as a discipline and that there is a need to clarify that it is not a sub-discipline of medicine.

Box 2 Defining characteristics of behavioral medicine

**Table:** 

1. Behavioral medicine is a *field*, and not a discipline. 2. Behavioral medicine’s uniqueness is based on *transcending disciplinary boundaries* to create new knowledge or practice. 3. Behavioral medicine is characterized by the *interaction of research* (development of knowledge) *and practice* (professional practice in clinical and public health). 4. Behavioral medicine is characterized by activities in three domains—*bio-behavioral mechanisms*, *clinical practice*, and *public health*—and the interaction of activities in these domains.	

##### Item 3. Behavioral Medicine: Research and Practice

There was consensus concerning the idea that behavioral medicine is comprised of both the development of knowledge and techniques (research) and the application of said knowledge and techniques (practice) (agreement 91%). Several respondents mentioned that the flow of knowledge between research and practice is not unidirectional; instead, it might be better to discuss “research-practice interactions,” in the sense that research and practice are linked and inform each other’s development. One respondent mentioned that research should provide evidence that is useful for practice.

##### Item 4. The Scope of Behavioral Medicine Extends from Bio-behavioral Mechanisms to Clinical Diagnosis and Intervention and to Public Health

Despite the consensus on scope (agreement 87%), there were several suggestions to improve the wording in order to avoid suggestion of a unidirectional relationship implied by the phrasing “extends from .. to ..,” instead emphasizing the interactions between bio-behavioral mechanisms, clinical diagnosis and intervention, and public health. There were single suggestions not to refer to clinical diagnosis; to de-emphasize public health or not to include public health at all; to add health services; to add sectors outside medicine and health care; and to add dissemination.

##### Item 5. Support and Facilitation of Collaboration Among Behavioral and Biomedical Disciplines

There was consensus to add facilitation of collaboration among behavioral and biomedical disciplines to the current tasks and goals of ISBM (agreement 96%). Several respondents mentioned that support and facilitation of collaboration among behavioral and biomedical disciplines could become the core feature and central part of ISBM’s identity, with great strategic potential. Specific suggestions for collaboration were given, such as arranging tracks at medical, psychological, and behavioral congresses.

##### Item 6. Collaboration Among Mono-disciplinary Organizations Interested in Activities at the Interface of Behavior and Biomedicine

Despite the consensus that ISBM could serve as an international platform for collaboration among mono-disciplinary organizations with interests at the interface of behavior and biomedicine (agreement 96%), there were several comments expressing doubts about the need to add this task: the current listing of tasks and goals may be sufficient, especially if one adds “support and facilitation of collaboration among behavioral and biomedical disciplines” (item 5). It was pointed out that ISBM already has a membership category for “specialized societies” (that is, mono-disciplinary organizations), but that there has not been much effort to set up collaborations with such societies.

##### Item 7. International Platform for Exchange of Activities Related to Clinical and Public Health Practice

There was consensus to add activities related to practice in clinical care and public health to ISBM’s current tasks (agreement 83%). It was mentioned that greater clarity is needed as to exactly how this could be achieved. Guidelines and policy/practice briefs, education and training (short professional courses, links to medical schools), and practice-related special interest groups were suggested as potentially fruitful activities.

##### Item 8. International Platform for Multidisciplinary Collaboration with Regard to Education and Training in Behavioral Medicine

The panel reached consensus on the idea that ISBM would serve as an international platform for multidisciplinary collaboration regarding education and training in behavioral medicine (agreement 96%). In fact, it was noted that education and training is already a task, and several respondents stated that there was a need to better emphasize this task.

##### Item 9. Name Change

In response to the question as to whether ISBM should consider a name change (for example, International Society of Behavioral Health and Medicine), no consensus was reached (agreement 48%). Respondents pointed out that the connotations of “behavioral health” vary strongly between countries; that “behavioral health” is difficult to translate in their language; that the term “behavioral medicine” has been used for some time and is generally accepted to include all aspects of the current definition; and a name change might be confusing. Other respondents supported the suggestion to add “health” to the name, to convey more explicitly ISBM’s broad and multidisciplinary community, and to emphasize that behavioral medicine is not a sub-discipline of medicine. Some respondents stated that national or regional societies can include behavioral health in their own name. Another suggestion was to include “science” in the name (for example, International Society of Behavioral Science in Health Care and Medicine).

Box 3 Proposal on the main tasks and goals of ISBM

**Table:** 

**Communication, collaboration, and liaison** • To support and facilitate communication and collaboration among behavioral and biomedical disciplines • To encourage the organization of scientific meetings related to behavioral medicine, at the national, regional, and international levels • To encourage the publication and dissemination of scientific information on behavioral medicine, with an emphasis on international impact • To maintain liaison with related scientific societies and networks, and with related international, professional, and policy organizations **Research** • To stimulate research in the field of behavioral medicine, with an emphasis on the international collaborative level **Education** • To stimulate education and training activities related to behavioral medicine, in particular the development of curricula, materials, workshops, and courses **Practice** • To raise the profile of behavioral medicine and to serve as an information source on activities in the field of behavioral medicine • To serve as an international platform for activities in clinical and public health practice • To stimulate the development of standards, guidelines, and practice/policy briefs on behavioral medicine **Support** • To encourage the formation of national or regional organizations for behavioral medicine • To support and recognize scientists working in behavioral medicine, with an emphasis on—but not limited to—professionals at an early stage of their career	

##### Additional Comments

A further suggestion was to add “enhancing visibility of the field to the general public” and “raising the profile of behavioral medicine, with a focus on public health policymakers and medical educators” to the current tasks.

#### The Core Group’s Considerations Following Round 1

##### Definition and Scope

In response to the suggestions from the Delphi panel, the core group proposed the following: adding an explanation of “behavioral knowledge” to the definition; combining psychological and social into “psychosocial” processes; adding economic processes; considering political processes as a category of societal processes; removing references to “techniques”; and adopting some of the proposed edits on the definition and scope.

##### Defining Characteristics

The core group noted consensus on four defining characteristics of behavioral medicine. *First*, behavioral medicine is a field and not a discipline. Behavioral medicine is a field arising from the collaboration of people with a scientific background in behavioral disciplines (concerned with psychosocial, societal, economic, cultural, existential, and environmental phenomena) and biomedical disciplines (concerned with phenomena in the physiological, pathological, or medical domain). *Second*, transcending disciplinary boundaries to create new knowledge or practice is a defining characteristic of behavioral medicine. The core group agreed that transdisciplinary and inter-professional collaboration is not exclusively about synthesizing different perspectives; instead, the intention is to transcend disciplinary boundaries to create new knowledge or practice. *Third*, behavioral medicine is characterized by the interaction of research (development of knowledge) and practice (professional practice in clinical and public health). Research and practice interact and can mutually inform each other’s development. *Fourth*, behavioral medicine is characterized by activities in three domains—bio-behavioral mechanisms, clinical practice, and public health—and the interaction of activities in these domains. The translation of findings in one of these domains into the other domains contributes significantly to the successful development of the field as a whole. There is a need for balanced development and representation of all three domains.

##### Main Tasks and Goals

The core group agreed that “support and facilitation of collaboration among behavioral and biomedical disciplines” could be a key point when defining ISBM’s main tasks and goals. This task can be combined with the current task of enhancing interdisciplinary communication. It was agreed that there is no need to separately mention “collaboration among mono-disciplinary organizations interested in activities at the interface of behavior and biomedicine.” However, it was stressed that collaboration with mono-disciplinary organizations should be strongly encouraged. The core group agreed to add “creating an international platform for activities related to clinical and public health practice” to the tasks and goals. It was agreed that although “education and training” is already included, there is a real need to emphasize this task. The core group also agreed to add “raising the profile of behavioral medicine” to the tasks and goals; target groups include public health policymakers, medical educators, the general public, and other relevant groups. Finally, the core group noted that it would not be possible to simply add new tasks and goals to the current ones (see Supplementary file 4), as this would result in a poorly structured text. They agreed on the need to rewrite the tasks and goals, and that the Delphi panel should be invited to rate the proposal on the tasks and goals.

##### Name Change

The core group acknowledged various pros and cons regarding a name change. The connotations of “behavioral health” vary between countries, and a name change might be confusing. On the other hand, adding “health” to the name could more explicitly convey ISBM’s broad and multidisciplinary community and emphasize that behavioral medicine is not a sub-discipline of medicine. Weighing these arguments, the core group proposed retaining the current name, but to encourage national and regional societies to select a name that reflects their needs and purposes, and to emphasize in communications that ISBM is a broad and multidisciplinary community. It was decided that the Delphi panel should be invited to rate the proposal not to change the society’s name, and to provide comments.

#### Round 2

The core group defined 3 items for discussion in round 2. Consensus was reached for all 3 items, with the percentage of agreement ranging from 81 to 96%. The ratings are listed in Supplementary file 3, Table S2. The panel provided further comments.

##### Item 1. The Definition and Scope of Behavioral Medicine, and Its Defining Characteristics

The panel reached consensus on the proposed definition, scope, and defining characteristics of behavioral medicine (agreement 92%). Respondents suggested further emphasizing “psychosocial” by referring to biomedical, psychosocial, and behavioral knowledge, or conversely, not to use the term “psychosocial” at all. Respondents mentioned that the definition is still difficult to remember and suggested shortening it. The panel suggested several edits to improve the wording.

##### Item 2. Tasks and Goals

The panel reached consensus on the proposed tasks and goals of ISBM (agreement 96%). Suggestions included explicitly adding collaboration with other organizations and providing more detail on “research.” The panel suggested several edits to improve the wording.

##### Item 3. Proposal Not to Change the Name

Although the consensus was not to change the society’s name (agreement 81%), several panelists emphasized the need to improve the society’s appeal to non-clinicians. Adding a tagline to the name was suggested as a means to convey additional information that is not immediately evident when hearing the society name. One suggestion was to include “science” in the society’s name. The need to clarify the distinction between behavioral medicine and other fields and disciplines was also mentioned. The panel suggested several edits to improve the wording.

#### The Core Group’s Considerations Following Round 2

The core group agreed to add collaboration with other organizations to the tasks and goals, and to make several edits to improve the wording. The core group decided to ask for the panel’s opinion on adding a tagline to the society name, and to request suggestions regarding a tagline. The group considered a discussion on the distinction between behavioral medicine and other fields and disciplines as beyond the scope of its mandate, but it was suggested that a follow-up project could focus on that distinction.

#### Round 3

The core group defined 1 item for discussion in round 3, on the need to add a tagline to the society’s name. Consensus was reached to add a tagline (agreement 88%). The suggestions and ratings for a specific tagline are listed in Supplementary file 3, Table S3.

#### The Core Group’s Considerations Following Round 3

The core group decided not to vote on a specific tagline, bearing in mind that this decision should be based on ISBM’s communication strategy and specialized expertise in communication. Instead, the tagline suggestions were forwarded to ISBM with a recommendation for further decision-making on this issue. In addition, the wording of the definition of behavioral medicine was improved, by splitting the first, rather long sentence into two sentences.

#### Round 4

One of the reviewers of the first version of the present paper expressed concern that the explanation of “behavioral knowledge” did not include behavioral processes, which resulted in a very broad definition. The core group agreed with this comment and proposed to amend the explanation of “behavioral knowledge” as follows: “behavioral knowledge refers to psychosocial, societal, economic, cultural, existential and environmental processes of health- and disease-related behavior,” with the understanding that health- and disease-related behavior refers to overt behavior (e.g., physical activity) as well as related cognition, emotion, and motivation. The panel reached consensus on this amendment (agreement 100%). The ratings are listed in Supplementary file 3, Table S4.

## Discussion

The international panel reached consensus on an updated definition and scope of behavioral medicine and its four defining characteristics, and on a proposal regarding ISBM’s main tasks and goals. The international panel consisted of experts in the field, representatives of ISBM member societies, ISBM’s early career network, and several emerging collaborative groups in the field of behavioral medicine. As the response rates and the degrees of consensus were high (well above 80% and well above the a priori defined criterion of 70%, respectively), there appears to be strong support for the present proposals.

The original definition of behavioral medicine by Schwartz and Weiss [1] and the scope of behavioral medicine in the ISBM Charter [5] are clearly still relevant. However, the explanation of “behavioral knowledge” is an important difference between the updated and original definition. The updated definition uses a broad understanding of “behavioral knowledge,” referring to psychosocial, societal, economic, cultural, existential, and environmental processes of health- and disease-related behavior, with the understanding that health- and disease-related behavior refers to overt behavior (e.g., physical activity) as well as related cognition, emotion, and motivation. Another difference is the emphasis on “research and practice.” The flow of knowledge between research and practice is not unidirectional, from research to practice. Instead, research and practice are linked, and they mutually inform each other’s development: research findings are implemented in clinical and public health practice, while experiences in practice lead to innovations in research. Other differences between the original and updated definitions include the addition of “health promotion” and “care” as areas where behavioral medicine is being applied, in addition to several rewordings. Finally, the definition in the ISBM Charter [5] refers to “etiology” as an area where behavioral medicine is being applied. In the updated definition, etiology was deleted because etiology is not an area where knowledge is being applied: etiology is not an intervention [7] (Supplementary file 1). This does not mean that etiology is not important. On the contrary, behavioral factors are very important in the etiology of disease: this is being studied under “bio-behavioral mechanisms.”

The four defining characteristics provide further clarification and explanation of the definition of behavioral medicine. Importantly, these four characteristics provide clear guidance on the tasks and goals of ISBM: (i) Behavioral medicine is a field (and not a specific discipline). Consequently, facilitating and promoting communication and collaboration between behavioral and biomedical disciplines is a key task of ISBM, and ISBM should promote communication and collaboration between these disciplines in research, education, and practice. (ii) In doing so, the focus should be on transcending traditional disciplinary boundaries to create new knowledge or practice, instead of just synthesizing different perspectives. (iii) Behavioral medicine is characterized by the interaction of research and practice. Consequently, ISBM should facilitate the exchange of information between research and practice. ISBM should also maintain links with related international, professional, and policy organizations, and it should serve as an international platform for exchange of activities in clinical and public health practice. (iv) Finally, behavioral medicine is characterized by activities in three domains: bio-behavioral mechanisms, clinical practice, and public health. Therefore, ISBM should encourage productive exchange and collaboration between these domains in its activities related to meetings and publications, research, education, practice, and support.

The diverging perspectives on the need for a name change did not come as a surprise: discussions on name changes tend to be long and difficult. However, it should be noted that this discussion touched upon crucial issues. Most importantly, transcending disciplinary boundaries is a key characteristic of behavioral medicine. In behavioral medicine, various disciplines interact on an equal footing; this is crucial for successful collaboration and the development of truly innovative research and practice. On the other hand, the connotations of “behavioral health” vary strongly between countries, which make it a less desirable term. While it has been clearly stated that “behavioral medicine” is not a sub-discipline within medicine, it seems important to add a tagline to the Society’s name to convey that information. In that respect, including terms such as “transcending disciplinary boundaries” and “health” in the tagline is key. Further decision-making could be embedded in a future ISBM communication strategy, elaborating on the various suggestions on a tagline generated by the panel (see Supplementary file 3, Table S3).

The discussion on the name change and on main tasks and goals both focused on ISBM. It should be noted, however, that these discussions have implications for the entire field of behavioral medicine, not only for ISBM. The name change would have applied to the entire field, not only the society. The tasks and goals are for ISBM as the umbrella organization in the field. Although it is a limitation of the present study that this broader perspective was not clearly mentioned in the discussion paper and the questions, it can be assumed that the panel was aware of this broader perspective.

The present study adds substantially to our earlier work [6, 7]. The explanation of “behavioral knowledge” has been improved; the interaction between research and practice has been emphasized, with research and practice mutually informing each other; and the four main characteristics of behavioral medicine have been clearly stated. The proposal on ISBM’s main tasks and goals was derived from the four main characteristics—this is entirely novel work. Finally, the definition, main characteristics, and main tasks and goals are all based on a solid international consensus obtained in a rigorous Delphi study, as opposed to informal consensus among a small group of authors [6, 7].

The field of behavioral medicine is evolving. Future developments may include greater emphasis not only on bioinformatics, artificial intelligence, and environmental science, but also on ethics and other disciplines. Collaboration with these disciplines may contribute to knowledge relevant to health and disease. In the future, the definition of behavioral medicine may have to be expanded to include these disciplines. Other future developments could include elaborating on the main characteristics of behavioral medicine (e.g., more emphasis on transcending disciplinary boundaries to create new knowledge or practice) or more emphasis on specific topics (e.g., prevention of infectious diseases).

Through a truly international discussion, consensus was reached on an updated definition and scope of behavioral medicine, and its defining characteristics, and on a proposal regarding ISBM’s main tasks and goals. The expanding reach of behavioral medicine renders crucial the need to develop a current and comprehensive definition, as well as characterization of scope and priorities. Our discussions have informed an update to the definition and scope of behavioral medicine and its defining characteristics, and provided guidance on ISBM’s main tasks and goals to help align with contemporary global perspectives. For the further development of the field of behavioral medicine and ISBM as the umbrella organization in that field, it is critical to generate clarity in terms of the definition and scope of our discipline and to delineate priorities for cultivating engagement and multidisciplinary collaboration at an international level. The work described herein provides a foundational step toward achieving these goals for ISBM and its member organizations.

## Electronic Supplementary Material

ESM 1 (DOCX 121 kb)
