# Supplementary material for: Definition and Characteristics of Behavioral Medicine, and Main Tasks and Goals of the International Society of Behavioral Medicine—an International Delphi Study
Source: Int J Behav Med. 2020 Sep 9;28(3):268–76. doi: 10.1007/s12529-020-09928-y (PMC8121730; doi:10.1007/s12529-020-09928-y)
Supplement: Supplementary file 1 — (DOCX 121 kb) [file 12529_2020_9928_MOESM1_ESM.docx]

*Version 11 June 2020*

**Definition and characteristics of behavioral medicine, and main tasks and goals of the International Society of Behavioral Medicine – an international Delphi study**

**Supplementary files**

**Supplementary file 1**

*Version 20 March 2019*

**ISBM Taskforce Definition, Goals and Organization**

*Definition of behavioral medicine, its defining characteristics and ISBM’s main tasks and goals (Discussion paper A)*

**Content**

- Background

- Definition

- Defining characteristics

- Main tasks and goals

- Another item for discussion

- Conclusion

- Appendix 1

- Appendix 2

[
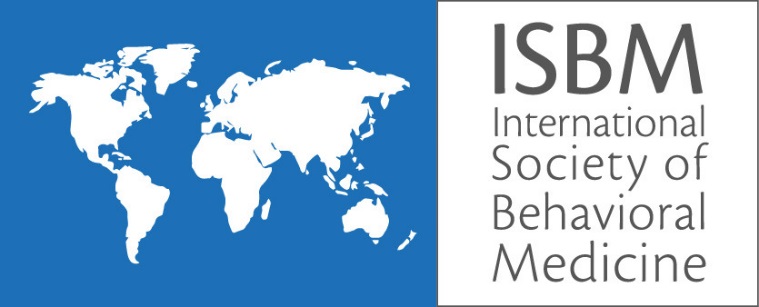
](https://www.isbm.info/)

**Background**

ISBM is facing a major paradox. Worldwide, the importance of behavior as a major determinant of health and disease is increasingly being recognized. The role of behavioral interventions in preventing and treating disease and promoting health is being emphasized. At the same time, ISBM and some of its member societies are facing stagnation: dwindling numbers of members in several ISBM member societies, the risk of several ISBM member societies fading away, confusion about the defining characteristics of behavioral medicine, difficulties in getting people to work on ISBM committees, and modest links to other professional societies are among the problems identified at the most recent Governing Council meeting (Santiago, November 2018).

The ISBM Board has decided to establish a Taskforce, to explore these problems and potential solutions**.** The goals of the Taskforce are to develop a proposal to update (i) the definition of behavioral medicine, and its defining characteristics; (ii) ISBM’s main tasks and goals; and (iii) the organization of ISBM. The present discussion paper concerns the first goals: the definition of behavioral medicine, its defining characteristics and its main tasks and goals (discussion paper A).

**Definition**

The original definition of behavioral medicine was developed at the Yale Conference on behavioral medicine and later published by Schwartz and Weiss (1). In its Charter, ISBM adopted a slightly different version of the definition, and ISBM defined the scope of behavioral medicine (2).

Recently, an update of the definition and scope of behavioral medicine was proposed (3). Several colleagues commented on this proposal (4-8), leading to a further revision of the definition, and invitation to contribute to the further discussion on the definition (9). The preliminary definition was discussed at the 2018 International Congress of Behavioral Medicine (10), as well as at several other occasions. The various definitions, the differences between these definitions and several considerations on potential updates are presented in Appendix A.

We propose that the original definition of behavioral medicine by Schwartz and Weiss and the definition of the scope in the ISBM Charter are remarkably adequate; only minor updates seem to be required. We propose the following updated definition and scope of behavioral medicine:

*Behavioral medicine can be defined as the field characterized by the collaboration among multiple disciplines concerned with the development and integration of behavioral and biomedical knowledge and techniques relevant to health and disease, and the application of this knowledge and these techniques to prevention, health promotion, diagnosis, treatment, rehabilitation, and care. The scope of behavioral medicine extends from bio-behavioral mechanisms, to clinical diagnosis and intervention, and to public health.*

An important clarification is that ‘behavioral knowledge ’refers to a wide range of knowledge, including knowledge on psychological, social, societal, cultural existential and environmental processes.

**Defining characteristics**

*(i) Behavioral medicine is a field, and not a discipline.*

Sommer (11) describes the difference between a discipline and a field as follows. “*Discipline*. Having the same root as disciple (one who learns), a discipline is a branch of instruction, or the educational aspect of a science or art. (…) Members of a discipline have been trained in departments associated with that discipline and study many different phenomena using a shared epistemology.” (p. 2) “*Field of study*. Based on a word meaning earth or land, a field is an area or sphere of action, operation, or investigation, a subject of activity or specialization. People in a field of study have been trained in various disciplines and professions but all focus on a common problem area.” (p.2 )

Behavioral medicine is the field characterized by the collaboration among behavioral and biomedical disciplines. Both ‘behavioral’ and ‘biomedical’ are broadly defined: behavioral disciplines include psychology, sociology, anthropology, philosophy, geography, etc.; biomedical disciplines include medicine, biomedical sciences, nursing, physiotherapy, etc. Each of these disciplines has its own body of knowledge. An essential characteristic of behavioral medicine is that is brings together multiple disciplines and their respective bodies of knowledge. Behavioral medicine is the field created by the collaboration among people with a scientific background in behavioral and biomedical disciplines.

Behavioral medicine is distinct from other fields and disciplines. The distinction between behavioral medicine and other fields and disciplines (such as health psychology, psychosomatic medicine, and mental health) has been extensively discussed elsewhere (3-9). Here, we refer to that discussion, and we focus on behavioral medicine’s key characteristics, tasks and goals.

*(ii) Behavioral medicine comprises both the development of knowledge and techniques (research) and the application of this knowledge and these techniques (practice).*

Both research and practice (clinical practice, health promotion and policies) are highly relevant. Research generates new knowledge and techniques, that can be applied in practice. Experience in practice leads to suggestions and questions, that can be studied in research. The interaction between research and practice is crucial for the development of behavioral medicine.

It should be acknowledged, though, that researchers and practitioners differ in their attitudes and needs. In a somewhat related field (education), Kezar (12) identified differences between practitioners and researchers with regard to what constitutes useful information (e.g., best practices, case scenario’s *versus* innovative theoretical perspectives, new data), what constitutes quality literature (e.g., relevance for day-to-day activity *versus* methodological rigor), useful formats (e.g., short, concise and summative *versus* detailed, detailed sections on theory and methodology), and significant issues and future directions.

Both researchers and practitioners are active in the field of behavioral medicine – and each group has specific attitudes and needs which should be addressed.

*(iii) The scope of behavioral medicine extends from bio-behavioral mechanisms, to clinical diagnosis and intervention, and to public health.*

The interaction among bio-behavioral mechanisms, clinical practice, and public health is crucial for the successful further development of behavioral medicine. Knowledge on bio-behavioral mechanisms is essential for the further development of clinical practice, and public health; interventions developed in clinical practice frequently are essential building blocks for public interventions; experiences in clinical practice and public health may provide clues to bio-behavioral mechanisms. The broad scope – extending from bio-behavioral mechanisms, to clinical diagnosis and intervention, and to public health - is the third defining characteristic of behavioral medicine.

**Main tasks and goals**

The main tasks and goals of ISBM are outlined in the current ISBM ‘statutes’ (see Appendix 2). These include development of national, regional, and specialized scientific (i.e., multinational) societies; interdisciplinary communication; international collaborative research activities; guidelines and standards for the implementation of behavioral medicine;

information dissemination and meetings; education and training; support and recognition.

The defining characteristics described above suggest that the main tasks and goals need to be expanded. There exist important tasks and goals, which are not mentioned in the current statutes.

*First*, behavioral medicine is the field characterized by the collaboration among behavioral and biomedical disciplines. This suggests that support and facilitation of multidisciplinary collaboration is a top priority. The current ISBM ‘statutes’ refer to several activities related to this task/goal, but fail to explicitly mention support and facilitation of multidisciplinary collaboration. *It is suggested here to add support and facilitation of collaboration among behavioral and biomedical disciplines as a high priority task/goal.*

*Second*, for the multidisciplinary collaboration to be successful, each of the disciplines needs to provide strong mono-disciplinary input into the multidisciplinary collaboration. Successful multidisciplinary collaboration requires strong mono-disciplinary input. Being interested in multidisciplinary collaboration is another essential requirement. It is suggested here that ISBM should explore how to encourage input from mono-disciplinary organizations into multidisciplinary activities. *ISBM could adopt the role of being the international platform for collaboration among mono-disciplinary organizations interested in activities at the interface of behavior and biomedicine, in addition to its traditional (and very valued) role in facilitating collaboration among multidisciplinary (member) organizations.* This would imply to develop and strengthen the links with primarily mono-disciplinary organizations such as organizations of physicians (cardiology, oncology, rheumatology, internal medicine etc.), health professionals (nursing, physiotherapy), professionals in public health, psychologists, and socio-cultural disciplines, in addition to the existing links with other multidisciplinary organizations (i.e., primarily the ISBM member organizations).

The broad scope of behavioral medicine – extending from bio-behavioral mechanisms, to clinical diagnosis and intervention, and to public health – implies that the international platform could involve not only clinical and public organizations, but also organizations involved in basic bio-behavioral research.

*Third*, behavioral medicine comprises both research and practice. Thus far, we have emphasized research: our journal and our congress are both highly respected and successful. Activities with regard to practice are somewhat limited. It is suggested here that ISBM could be much more active in exploring activities related to practice. This applies to both clinical care and public health. Examples include *guidelines and standards* (the collaboration with the European Cardiology Society is an outstanding example; other organizations are likely to be interested in ‘behavioral’ collaboration as well), *policy briefs*, etc. In addition, ISBM could facilitate collaboration and exchange with regard to *best practices,* for example in the field of e-health, behavior change techniques, health promotion etc. *ISBM could adopt the role of being the international platform for exchange of activities related to practice – both clinical practice and public health practice.*

*Fourth*, education and training is a related issue. ISBM could be more active in facilitating collaboration with regard to education and training in behavioral medicine. It seems that that there is huge demand for education and training in behavioral medicine. This suggest that ISBM can be especially valuable in this area. *ISBM could adopt the role of being the international platform for multidisciplinary collaboration with regard to education and training in behavioral medicine.*

**Another item for discussion**

Lau (6) called attention to the fact that a number of the ISBM member societies used behavioral health in naming their society, instead of behavioral medicine. ‘The choice may represent an attempt to clarify [that] their emphasis is on health-related issues and behaviors, and that their societies are open to both clinical and non-clinical members’ (6) (p. 15). It is important for ISBM to consider regionally appropriate titles for the field. ISBM needs to be appealing to both clinical and non-clinical people working in the field, in all regions of the world. Whether or not this implies a need to consider a name change needs further discussion (e.g., *International Society of Behavioral Health and Medicine.*

**Conclusion**

The proposed definition and description of defining characteristics of behavioral medicine highlight the need to consider expansion of the main tasks and goals of ISBM. We propose to continue the discussion on the tasks and goals of ISBM, although comments on the definition and the defining characteristics are welcome as well.

Authors:

JD and the other members of the core group of the ISBM Taskforce Definition, Goals and Organization

**Appendix 1.**

**Various definitions of behavioral medicine**

**1.** The definition of behavioral medicine as provided by **Schwartz and Weiss** (1):

*Behavioral Medicine is the interdisciplinary field concerned with the development and integration of behavioral and biomedical science knowledge and techniques relevant to health and illness and the application of this knowledge and these techniques to prevention, diagnosis, treatment and rehabilitation.*

**2.** The definition of behavioral medicine in the **ISBM Charter** (2):

*Behavioral medicine can be defined as the interdisciplinary field concerned with the development and integration of psychosocial, behavioral, and biomedical knowledge relevant to health and illness and the application of this knowledge to prevention, etiology, diagnosis, treatment, and rehabilitation.*

The ISBM Charter goes on to define the scope of behavioral medicine as:

*The scope of behavioral medicine extends from research efforts to understand fundamental biobehavioral mechanisms; to clinical diagnosis and intervention; to disease prevention and health promotion.*

**3.** The **differences** between the **Schwartz and Weiss** definition and the definition in the **ISBM Charter** are highlighted below:

Schwartz and Weiss: *Behavioral Medicine is the interdisciplinary field concerned with the development and integration of behavioral and biomedical science knowledge and techniques relevant to health and illness and the application of this knowledge and these techniques to prevention, diagnosis, treatment and rehabilitation.*

ISBM Charter: *Behavioral medicine can be defined as the interdisciplinary field concerned with the development and integration of psychosocial, behavioral, and biomedical knowledge relevant to health and illness and the application of this knowledge to prevention, etiology, diagnosis, treatment, and rehabilitation. The scope of behavioral medicine extends from research efforts to understand fundamental biobehavioral mechanisms; to clinical diagnosis and intervention; to disease prevention and health promotion.*

**4.** The **current proposal** for the definition of behavioral medicine:

*Behavioral medicine can be defined as the field characterized by the collaboration among multiple disciplines concerned with the development and integration of behavioral and biomedical knowledge and techniques relevant to health and disease, and the application of this knowledge and these techniques to prevention, health promotion, diagnosis, treatment, rehabilitation, and care. The scope of behavioral medicine extends from bio-behavioral mechanisms, to clinical diagnosis and intervention, and to public health.*

**5.** The **differences** between the **Schwartz and Weiss definition** and the **current proposal** are highlighted below:

Schwartz and Weiss: *Behavioral Medicine is the interdisciplinary field concerned with the development and integration of behavioral and biomedical science knowledge and techniques relevant to health and illness and the application of this knowledge and these techniques to prevention, diagnosis, treatment and rehabilitation.*

Current proposal: *Behavioral medicine can be defined as the field characterized by the collaboration among multiple disciplines concerned with the development and integration of behavioral and biomedical knowledge and techniques relevant to health and disease, and the application of this knowledge and these techniques to prevention, health promotion, diagnosis, treatment, rehabilitation, and care. The scope of behavioral medicine extends from bio-behavioral mechanisms, to clinical diagnosis and intervention, and to public health.*

**6.** The **differences** between the definition in the **ISBM Charter** and the **current proposal** are highlighted below:

ISBM Charter: *Behavioral medicine can be defined as the interdisciplinary field concerned with the development and integration of psychosocial, behavioral, and biomedical knowledge relevant to health and illness and the application of this knowledge to prevention, etiology, diagnosis, treatment, and rehabilitation. The scope of behavioral medicine extends from research efforts to understand fundamental biobehavioral mechanisms; to clinical diagnosis and intervention; to disease prevention and health promotion.*

Current proposal: *Behavioral medicine can be defined as the field characterized by the collaboration among multiple disciplines concerned with the development and integration of behavioral and biomedical knowledge and techniques relevant to health and disease, and the application of this knowledge and these techniques to prevention, health promotion, diagnosis, treatment, rehabilitation, and care. The scope of behavioral medicine extends from bio-behavioral mechanisms, to clinical diagnosis and intervention, and to public health.*

**7. Comments**

The current proposal for the definition is very close to original Schwarz and Weiss definition. In line with the ISBM Charter, we added the scope of behavioral medicine.

Instead of ‘interdisciplinary’, we used ‘characterized by the collaboration among multiple disciplines’, in order to avoid issues related to the terms multidisciplinary, interdisciplinary and transdisciplinary (13). We have gratefully accepted the suggestion to use ‘characterized by the collaboration among multiple disciplines’ (5, 9).

Elsewhere, we have clarified that ‘behavioral knowledge ’refers to a wide range of knowledge, including knowledge on psychological, social, societal, cultural, existential and environmental processes (3). This is an important clarification, that should be kept in mind when discussing the definition. The broad definition of ‘behavioral knowledge’ includes ‘psychosocial knowledge’, and thus there is no need to mention ‘psychosocial knowledge’ separately.

Behavioral medicine comprises research and the application of research findings. The Schwarz and Weiss definition emphasizes practical application by mentioning ‘techniques’. This is fully in line with the current emphasis on translational research (e.g. developing and applying behavior change techniques). We have chosen to explicitly mention ‘techniques’ in the definition.

We have previously argued that ‘etiology’ needs to be deleted from the areas where behavioral medicine is being applied (3). Behavioral medicine is being applied in areas such as prevention, health promotion, diagnosis, treatment, and rehabilitation. Etiology is in a different category, related to causal mechanisms, and does not fit here. It should be noted that the original Schwarz and Weiss definition refers to prevention, diagnosis, treatment and rehabilitation, and not to etiology.

We have argued that ‘health promotion’ and ‘care’ need to be mentioned as areas where behavioral medicine is being applied (3).

The ISBM Charter refers to ‘disease prevention and health promotion’. In this area, major developments have taken place (3). The role of health policy, health promotion, and health services as determinants of health and disease has been emphasized. In line with the World health Organization (14), we suggest to use ‘public health’ to refer to this area.

In conclusion, compared to the original Schwartz and Weiss definition and the definition in the ISBM Charter, only minor updates seem to be required. An important clarification is that ‘behavioral knowledge’ refers to a wide range of knowledge, including knowledge on psychological, social, societal, cultural, existential and environmental processes. With this clarification in mind, the current proposal for the definition and scope of behavioral medicine is:

*Behavioral medicine can be defined as the field characterized by the collaboration among multiple disciplines concerned with the development and integration of behavioral and biomedical knowledge and techniques relevant to health and disease, and the application of this knowledge and these techniques to prevention, health promotion, diagnosis, treatment, rehabilitation, and care. The scope of behavioral medicine extends from bio-behavioral mechanisms, to clinical diagnosis and intervention, and to public health.*

**Appendix 2.**

Statutes of the International Society of Behavioral Medicine, Article II, Section 2:

*The purpose of the ISBM e.V. is the promotion of science and research as well as undergraduate, graduate and professional education. The purpose is achieved in particular through the following projects:*

*- Development of national, regional, and specialized scientific (i.e., multinational) societies: To encourage formation of national or regional organizations of behavioral medicine, and to develop and maintain liaison with related professional organizations.*

*- Interdisciplinary communication: To encourage and coordinate communication and interaction among various health professionals including biomedical and behavioral science researchers and clinicians, without regard to specific discipline loyalties.*

*- International collaborative research activities: To stimulate cooperative research worldwide by promoting effective international communication between both individuals and national groups, through formal meetings, attendance and participation at other scientific meetings held by other societies in the field of behavioral medicine or related to behavioral medicine, collaborative undertakings, and awards for meritorious effort. Specific activities are undertaken to establish global networks including researchers and practitioners from low income and developing countries.*

*- Guidelines and standards for the implementation of behavioral medicine: To develop guidelines for implementation of behavioral medicine training and research activities at various levels in the health and behavioral sciences.*

*- Information dissemination and meetings: To serve as an information resource for behavioral medicine by means of facilitating access to scientific and professional journals, establishing worldwide computer based information systems, providing newsletters and other media to researchers, practitioners, public audiences (e.g. patients), and administrative authorities (especially of national health care systems), and encouraging scientific meetings/conferences.*

*- Education and training: To encourage education and training activities, in particular to assist in the preparation of curricula and course synopses for teachers in medical and behavioral science educational institutions, education and training materials (e.g., books, e-learning programs), and relevant teaching workshops and courses, where possible in connection with conferences and in cooperation with national, regional, and specialized scientific societies and organizations.*

*- Support and recognition. To support young scientists working in behavioral medicine. ISBM e.V. supports life-long learning of professionals in behavioral medicine and provides awards for meritorious effort. In regard to all activities the ISBM e.V. recognize the need to be sensitive to the cultural and scientific standards and practices in different parts of the world and to the fact that the scientific groups currently concerned with behavioral medicine vary in regard to research, education and clinical practice across nations.*

**References**

1. Schwartz GE, Weiss SM. Behavioral medicine revisited: an amended definition. J Behav Med. 1978;1(3):249-51.

2. International Society of Behavioral Medicine. Charter. www isbm info/about-isbm/charter/ [Internet]. 2019 15/01/2019.

3. Dekker J, Stauder A, Penedo FJ. Proposal for an Update of the Definition and Scope of Behavioral Medicine. Int J Behav Med. 2017;24(1):1-4.

4. Weiss SM. Proposal for an Update of the Definition and Scope of Behavioral Medicine: Commentary. Int J Behav Med. 2017;24(1):5-7.

5. Johnston M, Johnston D. What Is Behavioural Medicine? Commentary on Definition Proposed by Dekker, Stauder and Penedo. Int J Behav Med. 2017;24(1):8-11.

6. Lau JT. Commentary: Proposal for an Update of the Definition and Scope of Behavioral Medicine. Int J Behav Med. 2017;24(1):12-5.

7. Nater UM. Behavioral Medicine and Related Disciplines. Int J Behav Med. 2017;24(1):16-7.

8. Kawakami N. Reflections on the Proposed Definition and Scope of Behavioral Medicine. Int J Behav Med. 2017;24(1):18-20.

9. Dekker J, Stauder A, Penedo FJ. Defining the Field of Behavioral Medicine: A Collaborative Endeavor. Int J Behav Med. 2017;24(1):21-4.

10. International Congress of Behavioral Medicine. Meeting: Revising the definition of behavioral medicine: perspectives from ‘western’ and ‘non-western’ countries. 2018 [Available from: <https://issuu.com/zentidos/docs/icbm2018_program_book?e=5521120/65519331>.

11. Sommer R. Discipline and field of study: a search for clarification. Journal of Environmental Psychology. 2000;20(1):1-4.

12. Kezar A. Understanding the Research-to-Practice Gap: A National Study of Researchers' and Practioners' Perspectives. New Directions for Higher Education. 2000;110:9-19.

13. Choi BC, Pak AW. Multidisciplinarity, interdisciplinarity and transdisciplinarity in health research, services, education and policy: 1. Definitions, objectives, and evidence of effectiveness. Clin Invest Med. 2006;29(6):351-64.

14. World Health Organization. Public Health. www who int/trade/glossary/story076/en/ [Internet]. 2016.

**Supplementary file 2**

**Questions in Delphi round 1**

The questions refer directly to text in Discussion paper A.

1. Do you agree with the definition and scope of behavioral medicine, as stated above?

2. Do you agree with defining characteristic (i) Behavioral medicine is a field, and not a discipline ?

3. Do you agree with defining characteristic (ii) Behavioral medicine comprises both the development of knowledge and techniques (research) and the application of this knowledge and these techniques (practice)?

4. Do you agree with defining characteristic (iii) The scope of behavioral medicine extends from bio-behavioral mechanisms, to clinical diagnosis and intervention, and to public health?

5. Do you agree to add support and facilitation of collaboration among behavioral and biomedical disciplines as a high priority task/goal ?

6. Do you agree that ISBM could adopt the role of being the international platform for collaboration among mono-disciplinary organizations interested in activities at the interface of behavior and biomedicine, in addition to its traditional (and very valued) role in facilitating collaboration among multidisciplinary (member) organizations?

7. Do you agree that ISBM could adopt the role of being the international platform for exchange of activities related to practice – both clinical practice and public health practice?

8. Do you agree that ISBM could adopt the role of being the international platform for multidisciplinary collaboration with regard to education and training in behavioral medicine?

9. Do you think that ISBM should consider a name change ?

10. Do you have any other comments?

**Questions in Delphi round 2**

1. Your comments in round 1 have been summarized in the draft of a paper. Based on your comments, we have made some further improvements. Do you agree with the summary of your comments, the proposed definition and scope of behavioral medicine (see Box x), and its defining characteristics (see Box y)?

2. So far, the panel has discussed whether or not to add three tasks/goals to our current tasks/goals (as defined in our current Statutes). When we added the tasks/goals to the current ones, we noticed that this would result in a text with a very poor structure. We have edited the tasks and goals (see Box z in the paper). Do you agree with the tasks and goals as described in Box z ?

3. As you will see in the paper, we have no consensus on the need for a name change. After careful consideration, the core group proposes not to change the name, to encourage national and regional societies to have a name that reflects their needs and purposes, and to emphasize in communications that ISBM is a broad and multidisciplinary community. Do you agree with the proposal not to change the name?

**Question in Delphi round 3**

1. Do you agree to add a tagline to the Society’s name ?

If you agree, do you have suggestions on a tagline?

**Question in Delphi round 4^[[1]](#footnote-1)^**

1. Do you agree to amend the explanation of ‘behavioral knowledge’ as follows: ‘behavioral knowledge refers to psychosocial, societal, economic, cultural, existential and environmental processes of health- and disease-related behavior’?

**Supplementary file 3**

**Table S1. Summary of ratings in Round 1**

|  | Strongly agree | Agree | Neutral | Disagree | Strongly disagree | No response | Percentage agreement |
| --- | --- | --- | --- | --- | --- | --- | --- |
| Q 1 Definition and scope | 11 | 9 | 1 | 1 |  | 1 | 87% |
| Q2 Behavioral medicine is field | 15 | 7 | 1 |  |  |  | 96% |
| Q3 Research and practice | 16 | 5 | 1 | 1 |  |  | 91% |
| Q4 Mechanisms, clinical care and public health | 10 | 10 | 1 | 1 | 1 |  | 87% |
| Q5 Collaboration among behavioral and biomedical disciplines | 16 | 6 | 1 |  |  |  | 96% |
| Q6 Platform for collaboration among mono-disciplinary organizations | 16 | 6 |  | 1 |  |  | 96% |
| Q7 Platform for exchange of activities related to practice | 14 | 5 | 3 | 1 |  |  | 83% |
| Q8 Platform for multidisciplinary collaboration with regard to education and training | 16 | 6 | 1 |  |  |  | 96% |
| Q9 Name change | 5 | 6 | 6 | 3 | 2 | 1 | 48% |

N= 23

**Table S2. Summary of ratings in Round 2**

|  | Strongly agree | Agree | Neutral | Disagree | Strongly disagree | No response | Percentage agreement |
| --- | --- | --- | --- | --- | --- | --- | --- |
| Q1 Definition and scope | 19 | 5 |  | 2 |  |  | 92% |
| Q2 Tasks and goals | 16 | 9 |  |  |  | 1 | 96% |
| Q3 Proposal not to change the name | 14 | 7 | 2 | 3 |  |  | 81% |

N= 26

**Table S3. Summary of the ratings in Round 3.**

|  | Strongly agree | Agree | Neutral | Disagree | Strongly disagree | Abstain | Percentage agreement |
| --- | --- | --- | --- | --- | --- | --- | --- |
| Proposal to add a tagline | 11 | 10 | 2 |  | 1 |  | 88% |

N= 24

Suggestions for a tagline

- Transcending disciplinary boundaries for better health

- Dedicated to research, clinical practice and public health

- Dedicated to research, practice and public health

- Dedicated to research, clinical practice and public health in a global world

- Dedicated to interdisciplinary research and clinical practice for better health

- From biology via behavior to public health

- Seeking health and well-being from interdisciplinarity

- Education, research and practice

- Improving public health through education, research and practice

- Better through focus on behavior

- Better health through behavior change

- Interdisciplinary approach to promote health

- Utilizing behavioral science for maximizing health

- Connecting environment and health by behavior

- Science of behavior in health and medicine

- Behavioral science of health and medicine

**Table S4. Summary of ratings in Round 4.**

|  | Strongly agree | Agree | Neutral | Disagree | Strongly disagree | Abstain | Percentage agreement |
| --- | --- | --- | --- | --- | --- | --- | --- |
| Explanation of ‘behavioral knowledge’ | 18 | 9 |  |  |  |  | 100% |

N= 27

**Supplementary file 4**

Current statement on the purpose of ISBM

The purpose of ISBM is the promotion of science and research as well as undergraduate, graduate and professional education. The purpose is achieved in particular through the following projects:

- ***Development of national, regional, and specialized scientific (i.e., multinational) societies*:** To encourage formation of national or regional organizations of behavioral medicine, and to develop and maintain liaison with related professional organizations.

- ***Interdisciplinary communication:*** To encourage and coordinate communication and interaction among various health professionals including biomedical and behavioral science researchers and clinicians, without regard to specific discipline loyalties.

- ***International collaborative research activities*:** To stimulate cooperative research worldwide by promoting effective international communication between both individuals and national groups, through formal meetings, attendance and participation at other scientific meetings held by other societies in the field of behavioral medicine or related to behavioral medicine, collaborative undertakings, and awards for meritorious effort. Specific activities are undertaken to establish global networks including researchers and practitioners from low income and developing countries.

- ***Guidelines and standards for the implementation of behavioral medicine*:** To develop guidelines for implementation of behavioral medicine training and research activities at various levels in the health and behavioral sciences.

- ***Information dissemination and meetings*:** To serve as an information resource for behavioral medicine by means of facilitating access to scientific and professional journals, establishing worldwide computer based information systems, providing newsletters and other media to researchers, practitioners, public audiences (e.g. patients), and administrative authorities (especially of national health care systems), and encouraging scientific meetings/conferences.

- ***Education and training:*** To encourage education and training activities, in particular to assist in the preparation of curricula and course synopses for teachers in medical and behavioral science educational institutions, education and training materials (e.g., books, e-learning programs), and relevant teaching workshops and courses, where possible in connection with conferences and in cooperation with national, regional, and specialized scientific societies and organizations.

- ***Support and recognition*.** To support young scientists working in behavioral medicine. ISBM supports life-long learning of professionals in behavioral medicine and provides awards for meritorious effort. In regard to all activities ISBM recognizes the need to be sensitive to the cultural and scientific standards and practices in different parts of the world and to the fact that the scientific groups currently concerned with behavioral medicine vary in regard to research, education and clinical practice across nations.

1. This was called round 5, as another Delphi round on the future organization of ISBM was already called round 4. [↑](#footnote-ref-1)
